# Different histological classifications for Henoch-Schönlein purpura nephritis: which one should be used?

**DOI:** 10.1186/s12969-019-0311-z

**Published:** 2019-02-28

**Authors:** Marija Jelusic, Mario Sestan, Rolando Cimaz, Seza Ozen

**Affiliations:** 1 Department of Paediatrics, University Hospital Centre Zagreb, University of Zagreb School of Medicine, Zagreb, Croatia; 2 Meyer Children’s Hospital, University of Florence, Florence, Italy; 3 Department of Pediatrics, Hacettepe University, Ankara, Turkey

**Keywords:** Henoch-Schönlein purpura, IgA Vasculitis, Glomerulonephritis, Biopsy, Histology, Children

## Abstract

**Background:**

Nephritis is the most important chronic complication of IgA Vasculitis (IgAV)/Henoch-Schönlein purpura (IGAV/HSP) and thus the main prognostic factor of this most common childhood vasculitis. Since the prognosis and treatment selection depends on the mode of interpretation of biopsy material, in this manuscript we have presented several issues related to the uneven application of different histological classifications in IgAV/Henoch-Schönlein purpura nephritis (HSPN). The nephritis of IgAV/IGAV/HSP will be abbreviated as HSPN for this paper.

**Main body:**

In clinical practice we use different histological classifications for HSPN. It is not known which of these classifications best correlates with severity of renal disease and renal outcome in IgAV/IGAV/HSP. One of the major problem with existing histological classifications is that there is no consensus on the implementation of biopsy in the treatment of HSPN. There is a histologic classification system conventionally used in HSPN, of the International Study of Kidney Disease in Children (ISKDC). On the other hand there is the new classification system suggested for IgA nephropathy, the Oxford classification. The latter has been validated only in IgA nephropathy. There are also two further histologic classifications of Haas and Koskela that have been developed. Current treatment strategies in HSPN are not standardised nor predominantly based on histological classification.

**Conclusion:**

One of the possible solutions to problems related to the application of different histological classification in HSPN is the implementation of multicenter multinational prospective studies with joint collaboration between pediatric rheumatologists, nephrologists and nephropathologists to correlate the clinical features and outcome with the classification systems as well among the classifications. This classification should be the basis for the construction of guidelines for the treatment of patients with HSPN.

## Background

Henoch-Schönlein purpura (IGAV/HSP) or IgA vasculitis (IgAV) is the most frequent form of vasculitis in childhood, with an annual incidence of 13–20/100000 children under 17 years of age [1, 2]. The European League Against Rheumatism (EULAR), Paediatric Rheumatology International Trials Organization (PRINTO) and Paediatric Rheumatology European Society (PRES) (EULAR/PRINTO/PRES) defined the criteria for diagnosis of IgAV/IGAV/HSP [3].

Although the disease is typically self-limiting and the prognosis is largely good [4], renal involvement may advance and cause permanent organ damage in the form of severe nephritis (HSPN) [5] making the renal involvement of the disease the main prognostic factor [6, 7].

Since the confirmation of diagnosis of HSPN requires a biopsy of the kidneys, and histological classification of the obtained material has an important role to determine prognosis and therapy selection, in this review article we aim to describe existing problems related to the commonly used histological classifications for predicting outcome in HSPN.

## Main text

### Renal manifestations in IGAV/HSP

Renal involvement occurs in a variable proportion (20–60%) of children suffering from IGAV/HSP [2, 7, 8]. Vast majority of children with IGAV/HSP (97%) develop features of renal involvement within 6 months of disease onset, but sometimes HSPN may occur later [7, 9–11]. The renal disease spectrum ranges from urinary abnormalities (including hematuria or/and proteinuria) through nephritic and nephrotic syndrome to chronic renal failure. Renal involvement is typically mild and manifested only by pathological urine findings. The greatest number of patients with HSPN, about 50% of them, develop simultaneous hematuria and proteinuria [9, 12]. Nephritic or nephrotic syndrome occurs in about 20% of HSPN patients [9, 12]. Chronic renal failure in children was noted to occur in a wide range of proportions of patients, from 1 to 15% [7, 9, 13, 14]. Patients with both nephritic and nephrotic syndrome have the highest risk for progression to end-stage renal disease (ESRD): more than 50% of them will develop long-term renal impairment, compared to about 5% of patients with urinary abnormalities (hematuria and/or proteinuria) [9, 12].

### Different histological classifications for HSPN

Due to the fact that glomerulonephritis is the major cause of mortality and morbidity among children suffering from IGAV/HSP, the prognosis of the disease largely relies on the renal involvement of IGAV/HSP [6, 9]. A kidney biopsy is mandatory for the diagnosis of HSPN. However, its value as a predictor of the outcome is dependent on many variables, principally on the classification used. Today we use different histological classifications for HSPN and four of them, utilized in clinical practice and research studies, are described below.

The International Study of Kidney Disease in Children (ISKDC) classification [15, 16] is still the most frequently used for histological analysis of renal biopsy findings in HSPN [17] (Table 1). The basic morphological changes underlying to a big extent of this classification are crescents. These lesions, whose pathogenic mechanism of development is still not elucidated, are associated with capillary wall destruction due to fibrinoid necrosis, which is linked with endocapillary proliferation and infiltration of inflammatory cells, including macrophages and neutrophils [18]. According to ISKDC classification, renal biopsies can be categorized into one of six histological grades. The first five grades of histological lesions are based on the presence and number of crescents. Grade VI represents membranoproliferative like glomerulonephritis, characterized with changes in the mesangial elements and the glomerular capillary wall. Crescents seemed a promising prognostic factor according to earlier studies, but in later publications the correlation between crescents and outcome has not always been confirmed [10, 19–21]. Namely, studies have shown that even patients with low grade histologic lesions, in the absence of crescents, may develop chronic renal failure and that patients with higher histological stages may experience spontaneous healing of lesions [10, 20, 21]. Unfortunately this grading system, being grounded mostly on the state of glomeruli, only reflects active inflammation, in the same time neglecting vascular and tubulointerstitial changes [19, 22].

**Table 1:** The International Study of Kidney Disease in Children (ISKDC) classification of Henoch-Schönlein purpura nephritis (a modification according to ref. [16])

ISKDC grade	Description
Grade I	Minimal alterations
Grade II	Mesangial proliferation
Grade III	Proliferation or sclerosis with < 50% crescents ((a) focal or (b) diffuse)
Grade IV	Mesangial proliferation or sclerosis with 50–75%, crescents ((a) focal or (b) diffuse)
Grade V	Mesangial proliferation or sclerosis with > 75% crescents ((a) focal or (b) diffuse)
Grade VI	Membranoproliferative like glomerulonephritis

It was subsequently shown that the proportion of sclerotic glomeruli and interstitial fibrosis correlates better with the long-term outcome, leading to the idea that the Oxford classification, used in IgA nephropathy, could be used in predicting the progression of renal disease in HSPN [23–25]. This scoring system published in 2009 initially included four morphologic features: mesangial hypercellularity (M), endocapillary proliferation (E), segmental glomerulosclerosis/adhesion (S) and tubular atrophy/interstitial fibrosis (T) [26, 27]. These four parameters formed the MEST score. Crescents were not included in the first version of the classification. The working group has made recommendations for changes to the Oxford classification in 2016 and has proposed the addition of a C (crescent score) to MEST as well as revising the S score, considering the presence or absence of podocytopathic features (podocyte hypertrophy/tip lesions). The revised Oxford classification includes MEST-C score [28] (Table 2). However, the working group does not recommend the use of MEST-C score in HSPN since cases of patients with this condition were not included in the validation cohort.

**Table 2:** The Oxford classification of IgA nephropathy (according to ref. [28])

Histological variable	Description
Mesangial hypercellularity	M_0_ < 50% of glomeruli showing mesangial hypercellularity M_1_ > 50% of glomeruli showing mesangial hypercellularity
Endocapillary hypercellularity	E_0_ absent E_1_ present
Segmental glomerulosclerosis/adhesion	S_0_ absent S_1_ present presence or absence of podocyte hypertrophy/tip lesions in biopsies with S1
Tubular atrophy/interstitial fibrosis	T0 ≤ 25% of the cortical area affected by tubular atrophy or interstitial fibrosis T1 26–50% of the cortical area affected by tubular atrophy or interstitial fibrosis T2 > 50% of the cortical area affected by tubular atrophy or interstitial fibrosis
Cellular/fibrocellular crescents	C0 absent C1 present in at least one glomerulus C2 present in > 25% of glomeruli

Modified with permission from Elsevier Ltd.©

Histologic classification of IgA nephropathy of Haas is based on retrospective studies using development of ESRD as their primary endpoint. Haas reviewed the histologic features of 244 cases of IgA nephropathy and using this histologic classification found statistically significant correlation between IgA subclass and renal survival [29] (Table 3).

**Table 3:** Histologic classification of IgA nephropathy of Haas (according to ref. [29])

Haas classification	Description
Class I – Minimal histologic lesion	Glomeruli are normocellular, without segmental sclerosis, necrosis, or crescents.
Class II – Focal-segmental glomerulosclerosis (FSGS)-like	Glomeruli show focal and segmental sclerosis without mesangial or endocapillary hypercellularity, crescents, or necrosis.
Class III – Focal proliferative glomerulonephritis	50% or fewer of the glomeruli (not including globally sclerotic glomeruli) are hypercellular. This hypercellularity may be limited to mesangial areas, or include endocapillary hypercellularity, crescents, or necrosis.
Class IV – Diffuse proliferative glomerulonephritis	More than 50% of the glomeruli (not including globally sclerotic glomeruli) are hypercellular. This hypercellularity may be limited to mesangial areas, or include endocapillary hypercellularity, crescents, or necrosis.
Class V – Advanced chronic glomerulonephritis	40% or more of the glomeruli are globally sclerotic, and/or there is > 40% estimated tubular atrophy or loss in the cortex. If these criteria are met, the biopsy specimen is graded as class V regardless of other histologic features.

Modified with permission from Elsevier Ltd.©

The latest classification, developed by Koskela et al. in 2017., is the modified semiquantitative classification (SQC). It takes into account no less than 14 variables, evaluating both, the activity and chronicity components making it the most precise and most promising so far [30]. Glomerular, tubular, interstitial and vascular findings are scored and a maximum score is defined as total biopsy score. The scoring system also includes activity index, chronicity index, focal or diffuse mesangial proliferation. Koskela et al. evaluated comparatively the ISKDC and SQC classifications in a national cohort of paediatric HSPN patients. Unfortunately, the number of patients in the original study was far too low (53 patients) to properly validate the classification (Table 4). Although the research showed promising results, according to which the variables of SQC (the total biopsy score and activity index) are better and more sensitive than ISKDC grades in prediction of patient outcome in HSPN, a study of a greater number of patients is needed.

**Table 4:** Modified semiquantitative classification (according to ref. [30])

Modified SQC	Description	Score
Glomerular changes		
Lobulation	Active	0 - 1^a^
Mesangial proliferation	Active	0 - 1^a^
Crescents		
Cellular	Active	0 - 3^b^
Fibrous	Chronic	0 - 3^b^
Adhesions	Chronic	0 - 3^b^
Fibrinous thrombosis	Active	0 - 3^b^
Global sclerosis	Chronic	0 - 3^b^
Segmental sclerosis	Chronic	0 - 2^c^
Tubular changes		
Thickening of the basement membrane	Chronic	0 - 1^a^
Complete atrophy	Chronic	0 - 1^a^
Tubular dilatation	Active	0 - 1^a^
Interstitial changes		
Fibrosis	Chronic	0 - 1^a^
Inflammation OR periglomerular inflammation	Chronic	0 - 1^a^
Capillary changes		
Arteriosclerosis OR arterial inflammation	Chronic	0 - 1^a^
Focal or diffuse mesangial proliferation		0 for focal, 1 for diffuse

^a^0 = absent; 1 = present

^b^0 = 0% of glomeruli affected; 1 = 0–5% of glomeruli affected; 2 = 5–10% of glomeruli affected; 3= > 10% of glomeruli affected

^c^0 = 0% of glomeruli affected; 1 = 0–5% of glomeruli affected; 2= > 5% of glomeruli affected

Reproduced with permission from Springer Nature©

Table 5 summarize the most frequently used classifications for histological analysis of renal biopsy findings in HSPN in clinical practice around the world. From this table it is obvious the data about histologic classifications for HSPN from some parts of the world are scarce with surprisingly very few recent studies. It may be noticed there is a considerable number of articles in which different modified or local pathological scoring classification systems are used.

**Table 5:** Use of the ISKDC, the Oxford and other histological classifications for HSPN in different continents

Continent/Region	ISKDC classification Authors, ref.	Oxford classification Authors, ref.	Other classifications Name of classification, authors, ref.
Europe	Koskela et al. 2017 [30] Soylemezoglu et al. 2009 [21] Lucas Garcia et al. 2009 [47]	Mizerska-Wasiak et al. 2018 [41] Gülhan et al. 2015 [42] Calvo-Río et al. 2013 [43]	**Modified semiquantitative classification** Koskela et al. 2017 [30] **Local pathological scoring** Mohey et al. 2013 [48] Pillebout et al. 2002 [49] **The classification developed by Heaton** Altugan et al. 2009 [50] **Classification according to Emancipator** Rauta et al. 2002 [51] Coppo et al. 1997 [13]
North America	Tarshish et al. 2004 [31]	N/A	**The classification adapted from Andreoli and Bergstein** Foster et al. 2000 [52] **The classification developed by Heaton** Heaton et al. 1977 [53] **Meadow’s classification** Meadow et al. 1972 [54]
Latin America	Buscatti et al. 2018 [55] Fuentes et al. 2014 [56] de Almeida et al. 2007 [57]	N/A	N/A
Asia	Inagaki et al. 2018 [44] Fu et al. 2016 [58] Lim et al. 2016 [25]	Inagaki et al. 2018 [44] Xu et al. 2018 [17] Kim et al. 2014 [35]	**Japanese histologic classification** Inagaki et al. 2018 [44] **Scoring system described by Andreoli and Bergstein** Kanai et al. 2011 [59]
Australia	N/A	N/A	N/A
Africa	Naija et al. 2012 [60]	N/A	**Histologic classification of Haas** Mitchell et al. 2010 [61]

The correlation between clinical aspects and histopathologic findings in HSPN has been the subject of several studies. Firstly, it was found that the presence, type and number of glomerular crescents in a biopsy specimen has a large role in the prognostic process, detecting a poorer outcome in patients with more than 50% of crescents, which was the basis for forming the ISKDC classification [5, 9, 31, 32]. However, as noted above, other studies did not confirm this finding, perhaps due to an uneven distribution of crescents, too small biopsy specimen, the possibility of spontaneous healing of histological lesions or the fact that patients with crescents were treated more aggressively [10, 21, 33, 34]. Tubulointerstitial lesions were found to positively correlate with a more severe outcome [25, 35, 36]. Furthermore, chronic lesions alongside acute lesions were identified as predictors of poor prognosis [34], where the total biopsy score acquired with the modified SQC differentiated patients with a score of ≤10 points as patients with a favorable outcome and with a score of ≥11 as patients with a greater risk of renal deterioration [30].

### What are the problems with the existing classifications of HSPN?

It is necessary to point out several issues regarding the existing classifications of HSPN. Of all the classifications most commonly used in clinical practice as well as for research purposes, it is not known which one predicts the renal outcome in IGAV/HSP and has the strongest association with unfavorable outcome. There are no studies that compare all the most frequently used classifications. This certainly has implications for defining the standardised management since histology is an important point in deciding whom to treat and with which medications. Lupus nephritis is, like HSPN, another inflammatory renal disease, but here the treatment strategies are based on histological classification. This is possible since the classification system in use is associated with severity of renal disease and renal outcome [37, 38]. Although the evidence for treatment of childhood-onset systemic lupus erythematosus and lupus nephritis is limited and there is a need for further research, representative rheumatologists and nephrologists managed to reach a consensus [38].

An important problem of classifications for HSPN is that they have not been validated in (sufficiently large numbers of) children or have been validated only in IgA nephropathy. For example, in the recommendations for updating the Oxford Classification the working group clearly accentuated the necessity of inclusion of larger number of children to avoid inadequate statistical power due to the limited number of patients reaching end points [28]. To date only few studies compared clinical and histological features between adults and children with HSPN, and showed that there are differences between these age groups [39]. Although HSPN in adults is a rare disease, a recent study showed that renal pathological chronicity was more serious in adults than in children: approximately 30% of adults with HSPN develop chronic renal failure [39, 40].

Findings from research on IgA nephropathy have been applied also to patients with HSPN. For instance, due to shared clinical, immunological and histological findings between the two diseases, it has been suggested that the Oxford classification can be used in classifying HSPN and it has been used in clinical practice [17, 35, 41–45]. Although these two diseases share similar features, there are also clinicopathological differences that are not only the mere presence of extrarenal clinical signs in IGAV/HSP [12, 22]. The most important differences in the clinical presentation of the two diseases are age and clinical signs. While IGAV/HSP has a peak incidence in the age between 4 and 7 years, and HSPN is predominantly seen in childhood, this range for IgA nephropathy is between 15 and 30 years. HSPN patients more often present as nephrotic or nephritic syndrome, whereas in IgA nephropathy such initial presentation is rare. Furthermore, HSPN frequently has an acute onset and course while IgA nephropathy is usually chronic progressive renal disease that gradually leads to renal failure. The risk of ESRD is higher in children with HSPN than in children with IgA nephropathy. Histologically, it should be noted that necrotizing changes and fibrin deposits in the glomerulus as well as endocapillary proliferation and crescents formation are more common in HSPN than in IgA nephropathy. Pathohistological changes in IgA nephropathy are predominantly found in mesangium and gradually lead to glomerulosclerosis and interstitial fibrosis. The main mechanisms of damage in HSPN are glomerular endothelial lesions that may heal or progress to fibrosis with scarring and chronic kidney damage [22]. Although the similarities between HSPN and IgA nephropathy are numerous, differences in clinical manifestation, course of the disease and renal damage mechanisms raise the question of the efficacy of uniform treatment of these two conditions and the possibility of a different therapeutic response to the same treatment [22]. Therefore, all classifications for IgA nephropathy need to be validated in children with HSPN if we intend to apply them in clinical practice or trials.

## Conclusion

In conclusion, limited number of children with HSPN and relatively short periods of follow-up increase the need for multicenter multinational prospective studies with joint collaboration between pediatric rheumatologists and nephrologists. It is necessary to raise awareness of the fact that IGAV/HSP can cause renal complications on the long term, even years after episode of purpura or following resolution of urinary abnormalities [7, 9, 10]. Since, patients with IGAV/HSP without renal complications at diagnosis are followed by pediatric rheumatologists or nephrologists, shared efforts are needed to prevent these patients from being lost during the follow-up and to avoid overlooking the kidney disease. It is therefore very important for future studies to observe patients for a long period of time and at regular intervals to include those who have a silent and subtle, insidious progression of the disease as well as patients with relapses [6]. Of great significance remains the need to include in studies early stage patients to detect the risk factors for progression of disease and for chronic renal failure, and to avoid confounding factors caused by treatment and selection bias by including patients with severe renal impairment [17, 46]. Particular attention needs to be devoted to strengthening the cooperation of pediatric rheumatologists, pediatric nephrologists and nephropathologists in different centers aiming at facilitating international exchange of knowledge and data. Consequently, it is of crucial importance that pediatric rheumatologists are also involved in validation of histological classifications in children with HSPN.

Given the fact that not insignificant proportion of the total number of patients with IGAV/HSP will develop renal impairment, we hope that the efforts for finding proper histological classification in HSPN will result in success as has been the case with lupus nephritis.

## Abbreviations

ESRD: End-stage renal disease
HSP: Henoch-Schönlein purpura
HSPN: Henoch-Schönlein purpura nephritis
IgAV: IgA Vasculitis
ISKDC: International Study of Kidney Disease in Children
SQC: Modified semiquantitative classification
